# Static stretch affects neural stem cell differentiation in an extracellular matrix-dependent manner

**DOI:** 10.1038/srep08499

**Published:** 2015-02-17

**Authors:** Janahan Arulmoli, Medha M. Pathak, Lisa P. McDonnell, Jamison L. Nourse, Francesco Tombola, James C. Earthman, Lisa A. Flanagan

**Affiliations:** 1 Department of Biomedical Engineering, University of California, Irvine, 3210 Natural Sciences II, Irvine, CA 92697-2715, USA; 2 Sue & Bill Gross Stem Cell Research Center, University of California, Irvine, 845 Health Sciences Road, 3030 Gross Hall, Irvine, CA 92697-1705, USA; 3 Department of Physiology & Biophysics, University of California, Irvine, D380 Medical Sciences I, Irvine, CA 92697-4560, USA; 4 Department of Neurology, University of California, Irvine, 200 South Manchester Avenue Suite 206, Orange, CA 92868-4280, USA; 5 Department of Chemical Engineering and Materials Science, University of California, Irvine, 916 Engineering Tower, Irvine, CA 92697-2575, USA

## Abstract

Neural stem and progenitor cell (NSPC) fate is strongly influenced by mechanotransduction as modulation of substrate stiffness affects lineage choice. Other types of mechanical stimuli, such as stretch (tensile strain), occur during CNS development and trauma, but their consequences for NSPC differentiation have not been reported. We delivered a 10% static equibiaxial stretch to NSPCs and examined effects on differentiation. We found static stretch specifically impacts NSPC differentiation into oligodendrocytes, but not neurons or astrocytes, and this effect is dependent on particular extracellular matrix (ECM)-integrin linkages. Generation of oligodendrocytes from NSPCs was reduced on laminin, an outcome likely mediated by the α6 laminin-binding integrin, whereas similar effects were not observed for NSPCs on fibronectin. Our data demonstrate a direct role for tensile strain in dictating the lineage choice of NSPCs and indicate the dependence of this phenomenon on specific substrate materials, which should be taken into account for the design of biomaterials for NSPC transplantation.

Stem cells are the only cells in the body capable of indefinite self-renewal and differentiation into various cell types. *In vivo*, they reside within specific microenvironments, or niches, that contain various chemical and physical signals affecting cell function. CNS neural stem cells are present in the fetal as well as adult brain and are multipotent, thus able to differentiate into neurons, astrocytes and oligodendrocytes^1^. During development, embryonic neural stem cells form the neural tube, which then gives rise to the brain and spinal cord. Throughout life neural stem cells are present in the subventricular zone (SVZ) of the lateral ventricles and dentate gyrus (DG) of the hippocampus. Since the discovery of stem cells, much work has focused on studying the effect of the chemical environment (soluble growth factors, chemokines, morphogens, etc.) on their behavior. However, effort recently has shifted to studying the effects of the physical microenvironment on stem cell behavior.

Mechanotransduction describes the process by which cells convert physical stimuli into chemical or electrical responses. Various mechanical factors of the cellular microenvironment, both passive and active, can greatly influence the maturation and shape of cells, tissues, and organs under both physiological and pathological conditions (Fig. 1)^2^. In particular, substrate elasticity, or stiffness, is a passive mechanical cue that has been well studied and greatly impacts stem cell fate during differentiation. For example, human mesenchymal stem cells (hMSCs) express markers of either bone, muscle, or brain cells when differentiated on hard (~25–40 kPa), medium (~5–20 kPa), or soft (~0.1–1 kPa) substrates, respectively^3^. NSPC fate is also strongly influenced by substrate stiffness in a range physiologically relevant for CNS tissue^4^, such that softer substrates (<1 kPa) induce neuronal differentiation while stiffer substrates (>1 kPa) encourage generation of astrocytes^5^,^6^,^7^.

Stem cells also encounter many active mechanical forces that can affect their differentiation (Fig. 1). Uniaxial cyclic strain increases the expression of smooth muscle markers from hMSCs^8^,^9^,^10^. This active mechanical stimulus coordinates with TGF-β, a soluble factor that also induces smooth muscle markers in these cells, to affect MSC differentiation^10^. Equibiaxial static strain of hMSCs cultured in osteogenic conditions causes increased cell proliferation and production of vascular endothelial growth factor (VEGF) via ERK and p38 mitogen-activated protein kinase pathways^11^. Additionally, active mechanical stimuli affect pluripotent stem cells since local cyclic stresses lead to downregulation of the undifferentiated stem cell marker Oct3/4 in mouse embryonic stem cells^12^. Furthermore, active forces imparted by the microenvironment may impact NSPC differentiation *in vivo*^13^. In normal CNS development, tissue folding and cell sheet movements create local physical stresses on endogenous NSPCs^14^,^15^. In cases of CNS damage such as traumatic brain injury (TBI), there is acute physical straining of brain tissue that has been modeled *in vitro* via application of equibiaxial stretch, which significantly affects function of neurons and glia^16^. There has been little work investigating the influence of mechanical stretch on NSPC differentiation into the three cell types of the CNS, but gradual mechanical stretching enhances neurite elongation and maturation of neurons derived from adult rat hippocampal NSPCs^17^. Since mechanical forces are at play during development and in cases of trauma, it is important to determine their effects on NSPC differentiation.

## Results

### Static stretch decreases oligodendrocyte differentiation from mNSPCs

**Figure 2:**
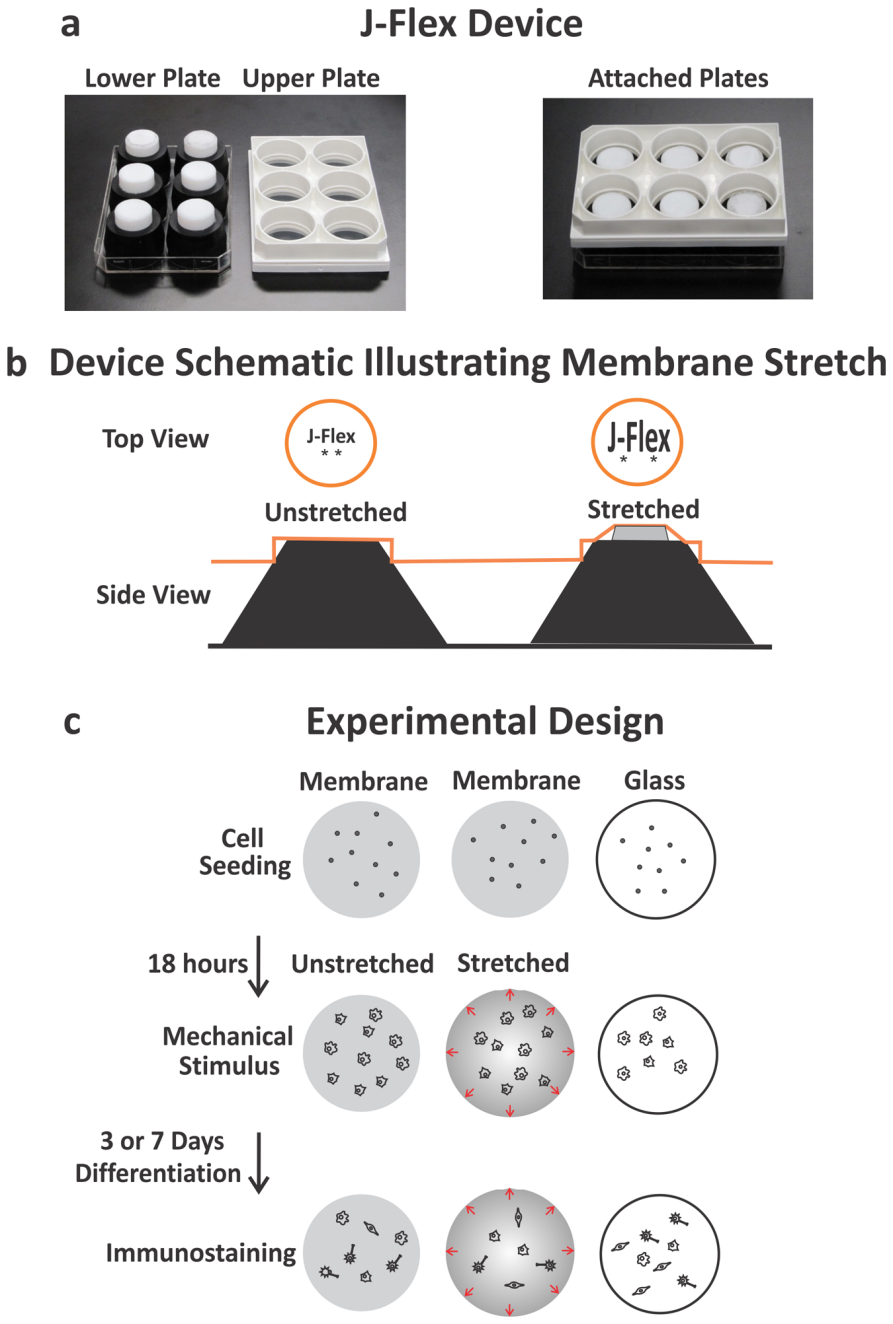
Induction of 10% equibiaxial static stretch to adhered NSPCs via the J-flex device. (a) J-Flex device: white polytetrafluoroethylene (Teflon) disks (25 mm diameter) attached to black rubber corks (lower plate) press fit into a Flexcell Bioflex plate (standard 6-well size) with silicone elastomer membranes (upper plate). When the two plates are attached, a 10% equibiaxial strain is induced on the silicone elastomer membranes (attached plates). Rubber bands (not shown) were used to keep the plates firmly press-fit. (b) Device schematic illustrating membrane stretch: top and side views of the membrane (orange circles in top view; orange line in side view), cork (black) and Teflon disk (grey) when the plates are attached. The stretched configuration shows displacement (10% equibiaxial) of two markings on the membrane in the top view and the corresponding setup with the cork and disk in the side view (not to scale). (c) Experimental design: mNSPCs were seeded on laminin-coated surfaces (membranes and glass) for 18 hours in proliferation conditions, followed by application of mechanical stimulus (stretched group only) after removal of growth factors (differentiation conditions) to assess formation of neurons (3 days), astrocytes (7 days), or oligodendrocytes (5 or 7 days) by immunostaining post-differentiation.

**Figure 3:**
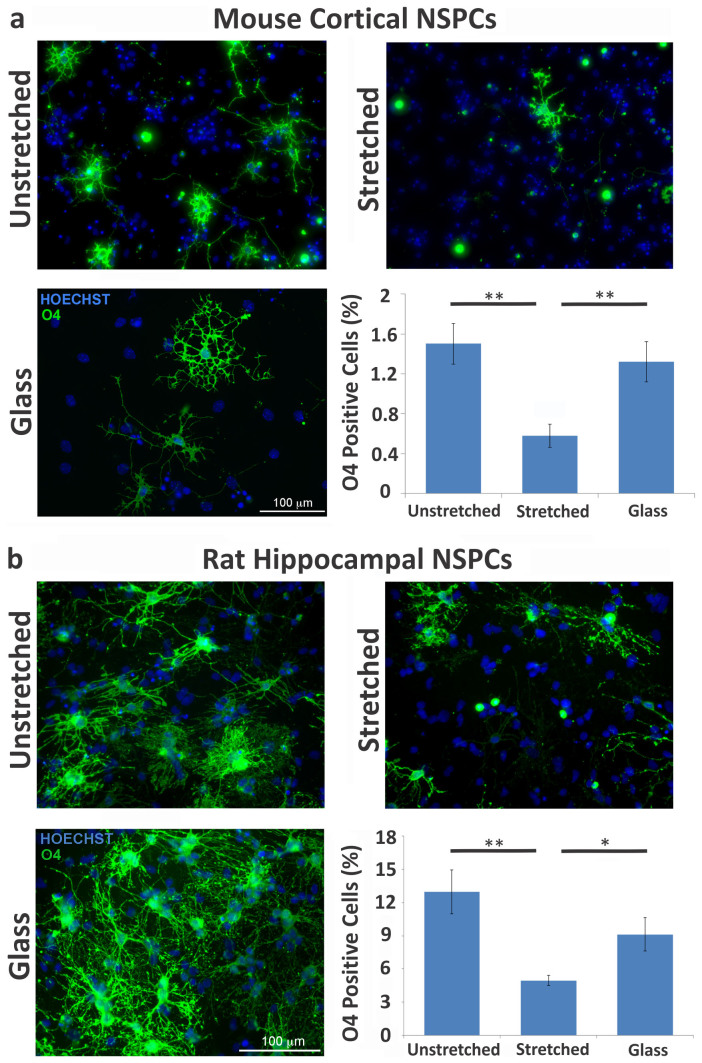
Stretch inhibits mNSPC differentiation into oligodendrocytes. (a) Mouse cortical NSPCs and (b) rat hippocampal NSPCs differentiated on unstretched and stretched membranes or glass were stained with oligodendrocyte cell surface marker O4 and Hoechst for nuclear DNA. Static stretch reduces oligodendrocyte differentiation regardless of cell source. (a) P = 0.0003 (unstretched vs. stretched), P = 0.002 (glass vs. stretched). (b) P = 0.001 (unstretched vs. stretched), P = 0.016 (glass vs. stretched). **P < 0.01, *P < 0.05. Error bars represent SEM. N = 3 independent biological repeats.

We tested whether an active mechanical stimulus alters NSPC differentiation by delivering a 10% static equibiaxial strain to cells via laminin-coated silicone elastomer membranes using a custom-built device, the J-Flex (Fig. 2a, b). We utilized mouse NSPCs (mNSPCs) derived from the embryonic cortex and quantified differentiation into neurons, astrocytes, and oligodendrocytes (Fig. 2c). We found no direct effect of static stretch on the differentiation of mNSPCs into neurons or astrocytes, which will be discussed further in a later section. However, oligodendrocyte differentiation was markedly affected. Specifically, generation of oligodendrocytes was significantly decreased on stretched compared to unstretched membranes, as illustrated by the number of cells positive for either the more mature oligodendrocyte marker O4 (Fig. 3a) or a marker of cells at an earlier stage of oligodendrocyte differentiation, platelet-derived growth factor receptor alpha (PDGFR-α) (Fig. S1). In fact, a single static stretch applied at the onset of differentiation and maintained for several days induced a 2.6-fold reduction in O4-positive oligodendrocytes and a 3.2-fold reduction in earlier stage oligodendrocytes detected by PDGFR-α (Figs. 3a and S1). Stretching the membranes increased membrane stiffness, which was controlled for by seeding and differentiating cells on membranes already undergoing stretch (pre-stretched condition) so that cells encountered the same stiffness as the stretched membranes but did not experience the stretch stimulus. This control distinguished the effects of stretch and stiffness. Oligodendrocyte differentiation was significantly higher on pre-stretched than on stretched membranes (O4-positive cells pre-stretched: 2.6 ± 0.4% and stretched: 0.6 ± 0.1%; error represents SEM), showing that the stretch-induced decrease in oligodendrocyte generation was not due to a stiffness change in the membrane. The observed decrease in oligodendrocyte differentiation in response to stretch was also not due to a significant effect on the adhesion, proliferation, or survival of cells on stretched membranes since there was no difference in total cell number counts on unstretched and stretched membranes (Fig. S2a). A single static stretch stimulus reduces the generation of oligodendrocytes from embryonic mNSPCs, and this effect is not due to either a stiffness change in the membrane or a change in the total cell number in the stretched condition.

We performed analogous experiments with adult rat hippocampal NSPCs to test whether the effects of stretch on oligodendrocyte generation are relevant to adult NSPC populations. A single static stretch induced a remarkably similar 2.6-fold decrease in the production of O4-positive oligodendrocytes from these cells, indicating their sensitivity to stretch (Fig. 3b). As seen with mNSPCs, the effect of stretch was not due to a stiffness change since oligodendrocyte differentiation was significantly higher on pre-stretched membranes than stretched membranes (O4-positive cells pre-stretched: 8.1 ± 1.1% and stretched: 5.0 ± 0.4%, p < 0.05; error represents SEM) nor to changes in the total cell number since this measure was equal on unstretched and stretched membranes (total cell number unstretched: 572 ± 57.4 and stretched: 546 ± 80.2, p = 0.792; error represents SEM).

### Laminin, but not fibronectin, plays a role in stretch-mediated oligodendrocyte differentiation

**Figure 4:**
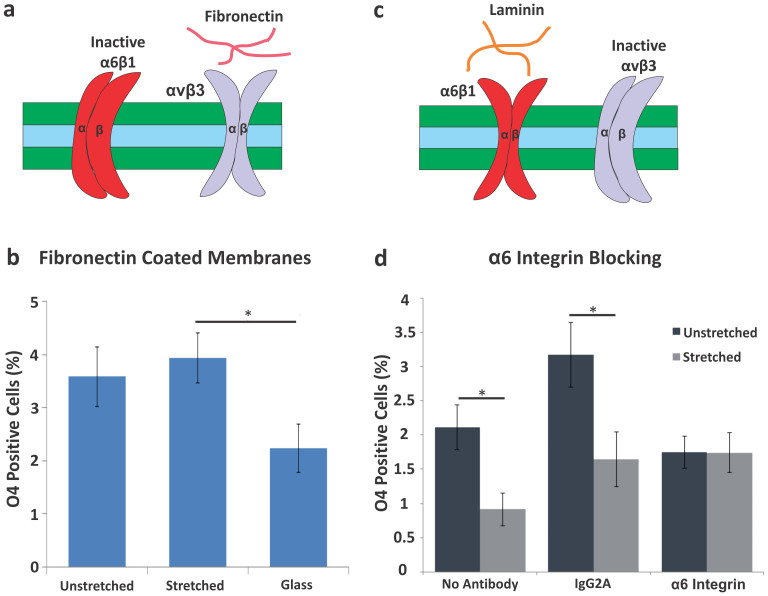
ECMs and integrins regulate mNSPC differentiation in response to static stretch. (a) Cells in the oligodendrocyte lineage express the laminin-binding integrin α6β1 but are driven to proliferate by fibronectin via the αVβ3 integrin^19^. (b) Delivery of a static stretch to mNSPCs on fibronectin does not affect differentiation into oligodendrocytes (no significant difference between unstretched vs. stretched group) although there is a difference between the stretched and glass groups. *P < 0.05. P = 0.013 (stretched vs. glass). Error bars represent SEM. N = 3 independent biological repeats. (c) Oligodendrocyte lineage cells can bind laminin through the α6β1 integrin to affect differentiation^20^. (d) Inhibiting α6 integrin with a function-blocking antibody negates the effect of stretch on oligodendrocyte differentiation from mNSPCs as shown by a lack of decreased O4 expression for cells on stretched membranes compared to those on unstretched membranes treated with α6 integrin antibody. *P < 0.05, P = 0.013 (no antibody). P = 0.025 (IgG2A). Error bars represent SEM. N = 3 independent biological repeats.

To investigate the mechanism behind the stretch-induced reduction in oligodendrocyte generation, we tested whether the specific type of ECM coating on the silicone elastomer membranes played a role. We found previously that human and mouse NSPCs cultured on laminin exhibited enhanced migration and differentiation into neurons and astrocytes in comparison to cells cultured on fibronectin or Matrigel^18^. Furthermore, ECMs regulate oligodendrocyte development since fibronectin stimulates proliferation pathways while laminin has been linked to survival and differentiation within this lineage^19^,^20^,^21^. To test the role of ECMs in the stretch-dependent reduction of oligodendrocyte generation from mNSPCs, we performed stretch experiments analogous to those shown previously on laminin (Fig. 3), but instead used fibronectin as an ECM (Fig. 4a, b). We found no effect of stretch on generation of oligodendrocytes from mNSPCs on fibronectin-coated membranes as illustrated by the similar percentages of O4-expressing cells in the unstretched and stretched groups (Fig. 4b). There was a significant difference between the stretched and glass groups, which is not easy to attribute to effects of either stretch or stiffness since differences were not significant between unstretched and stretched or unstretched and glass groups. The lack of an effect of stretch on fibronectin-coated membranes clarifies that the stretch-induced decrease in oligodendrocyte differentiation is specifically related to the cellular interaction with laminin and therefore suggests the involvement of laminin-binding integrins.

### E12.5 mNSPCs express functional α6 laminin-binding integrin

Cells bind to ECMs via integrins, which are transmembrane protein heterodimers consisting of α and β subunits. Integrins act as mechanosensors by forming bridges between the ECM and intracellular focal adhesions that connect to the actin cytoskeleton. Mechanical forces affect stem cells by altering the position and conformation of specific ECM components (such as laminin and fibronectin), thus triggering integrin-dependent signaling pathways to stimulate focal adhesion assembly^22^. Integrins have been well studied in the oligodendrocyte lineage and the laminin-binding integrin α6β1 is involved in oligodendrocyte differentiation and is exclusive to laminin without promiscuous binding to other ECMs^19^,^20^,^21^,^23^,^24^. We showed previously that human NSPCs express α6β1 and its function is critical for migration on laminin^18^. We therefore used a migration assay and a function-blocking antibody against the laminin-binding α6 integrin to discern whether mNSPCs also express functional α6β1 integrin. Treatment of mNSPCs with the α6 integrin function-blocking antibody did not appear to alter adhesion of the cells to laminin, which was based on visual inspection indicating no increase in non-adherent cells in wells treated with α6 antibody compared to controls. However, migration of cells treated with α6 antibody was significantly decreased compared to that of cells treated with IgG2A isotype control antibody or no antibody control (Fig. S3). These results indicate the expression of functional laminin-binding α6 integrins on mNSPCs.

### Blocking laminin-binding α6 integrin affects stretch-mediated reduction of oligodendrocyte differentiation

In order to test whether the α6 integrin is involved in the stretch-induced reduction of oligodendrocyte differentiation from mNSPCs on laminin, the α6 integrin function-blocking antibody was incubated with the cells at the onset of differentiation and stretch. Untreated and IgG2A isotype control antibody treated mNSPCs both revealed a decrease in oligodendrocyte differentiation with the stretch stimulus (Fig. 4d). Conversely, treatment of mNSPCs with α6 integrin blocking antibody abrogated the effect of stretch as evidenced by the lack of a marked decrease in generation of O4-positive oligodendrocytes with the application of stretch (Fig. 4d). While there was no significant difference in the generation of oligodendrocytes from cells on unstretched membranes in the no antibody control and α6 integrin antibody groups, there was a significant difference between the IgG2A control antibody and α6 integrin antibody unstretched membrane groups (p < 0.05). This brings up the possibility that blocking α6 integrin generally reduces oligodendrocytes, which would not be surprising given the role of this integrin in oligodendrocyte differentiation^20^. Alternatively, the IgG2A control antibody may be increasing oligodendrogenesis through an unknown mechanism. In either case, disrupting the function of the α6 integrin blocks the effect of stretch on oligodendrocyte differentiation, which does not occur with the controls. This suggests that the stretch-induced reduction of oligodendrocyte differentiation on laminin ECM may be mediated by the laminin-binding α6 integrin on mNSPCs.

### Distinct effects of static stretch and substrate stiffness on mNSPC differentiation

**Figure 5:**
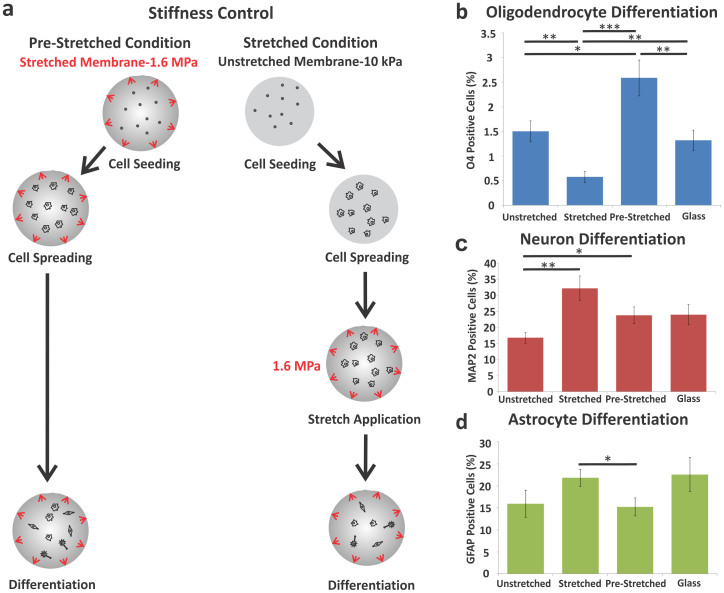
Effects of stretched membrane stiffness on NSPC differentiation. (a) Pre-stretched membranes were seeded with mNSPCs when the J-Flex device was already in place in order to control for the stiffness increase associated with the application of stretch at the onset of differentiation. Application of stretch to the membranes is denoted by red arrows. (b) Increase in stiffness upregulates oligodendrocyte differentiation as illustrated by the difference in percentage of O4-positive cells on pre-stretched vs. unstretched membranes. P = 0.0003 (unstretched vs. stretched). P = 0.01 (unstretched vs. pre-stretched). P = 1.9E−0.6 (pre-stretched vs. stretched). P = 0.002 (glass vs. stretched). P = 0.003 (glass vs. pre-stretched). (c) Stiffer membranes promote differentiation of more neurons from mNSPCs as demonstrated by significantly more MAP2-positive cells on pre-stretched or stretched membranes compared to cells on unstretched membranes. P = 0.002 (unstretched vs. stretched). P = 0.04 (unstretched vs. pre-stretched). (d) Negligible effects of stiffness or stretch on astrocyte generation as shown by minimal differences in GFAP-positive cells between stretched and pre-stretched against unstretched groups. P = 0.03 (pre-stretched vs. stretched). *P < 0.05, **P < 0.01, ***P < 0.0001. Error bars represent SEM. N = 3 independent biological repeats.

To test whether the observed stretch effect on differentiation is related to the increased stiffness of stretched membranes, we compared oligodendrocyte, neuron, and astrocyte generation on laminin-coated stretched and pre-stretched membranes that do not differ in stiffness (Fig. 5a). The stiffness of the pre-stretched control membrane matches that of the stretched membrane without delivering a stretch stimulus to the cells. NSPCs differentiated on pre-stretched membranes generated significantly more oligodendrocytes than cells on either unstretched or stretched membranes or glass (Fig. 5b and S4). Thus, increasing membrane stiffness from 10 kPa to 1.6 MPa induces more oligodendrocytes, but a single stretch at the onset of differentiation completely overcomes the stiffness stimulus and leads to a decrease in oligodendrocyte formation (stretched compared to pre-stretched membranes). Laminin molecules on the pre-stretched membrane might be altered by the stretch, which would be a confounding variable since cells are plated on the membrane after stretch application. To control for this, we stretched the membranes then coated with laminin to avoid perturbation of the laminin molecules (prestretched-then-coated). There were no differences in oligodendrocyte differentiation on pre-stretched [laminin coating → stretch → cell seeding] compared to prestretched-then-coated membranes [stretch → laminin coating → cell seeding] (O4-positive cells pre-stretched: 0.9 ± 0.3% and prestretched-then-coated: 1.1 ± 0.3%; error represents SEM). As observed with the stretch stimulus, the stiffness effect on oligodendrocyte generation was not due to a significant change in the adhesion, proliferation, or survival of cells on pre-stretched membranes since there was no difference in total cell number counts on unstretched and pre-stretched membranes (Fig. S2a). Collectively, these data indicate that two mechanical factors, stiffness and stretch, have significant and varying effects on differentiation of oligodendrocytes from NSPCs.

Static stretch had negligible effects on differentiation of neurons from mNSPCs. Neuronal differentiation, as shown by MAP2-positive cells, significantly increased in the stretched and pre-stretched conditions as compared to the unstretched condition, suggesting a potential stiffness effect (10 kPa unstretched vs. 1.6 MPa stretched and pre-stretched) but no influence of stretch (Fig. 5c and S4). Similar results were obtained when neurons were co-stained with two neuronal markers, MAP2 and doublecortin (DCX), which detects immature neurons (Fig. S5). The total number of cells on unstretched and pre-stretched membranes and glass did not differ, but there were significantly fewer cells on the stretched membranes (Fig. S2b). Thus, the significant increase in the percentage of neurons on pre-stretched membranes compared to the unstretched membranes may indicate an effect of stiffness on neuronal differentiation, but the increased neurogenesis on stretched membranes could indicate a stiffness effect or be due to reduced cell densities on those membranes.

We found no clear effect of static stretch on astrocyte generation from mNSPCs. We observed neither a stretch nor stiffness-dependent effect on astrocyte differentiation from mNSPCs since the stretched group demonstrated a significant increase in GFAP-positive staining in comparison with the pre-stretched group, but not against the unstretched control group (Fig. 5d and S4). Furthermore, there was no difference between the glass and stretched membrane group, and the percentages of GFAP-expressing cells across all conditions were quite similar (less than 1.5 fold difference between any of the groups). The total number of cells on membranes was very similar, but there was a significant difference between the unstretched and glass groups (Fig. S2c). However, since there was no difference in astrocyte differentiation between these groups (Fig. 5d), the data suggest that there was not a significant effect of cell adhesion, survival, or proliferation on the generation of astrocytes on the different surfaces. These findings indicate no significant effects of stretch or stiffness on astrocyte differentiation from mNSPCs in our system.

## Discussion

We found a single static stretch significantly and specifically impacts NSPC differentiation along the oligodendrocyte lineage, an effect that is dependent on the ECM composition. Oligodendrocyte differentiation is affected by static stretch on laminin but not fibronectin, and is potentially mediated by the α6 laminin-binding integrin. The interaction of stretch and specific ECM components may play a pivotal role in NSPC differentiation *in vivo.* The developing brain is rich in laminin^25^, and laminin is expressed in the SVZ and DG in the adult brain, which are two NSPC niches that remain throughout life^26^,^27^. Active mechanical forces induced during development by robust cell movements and tissue folding and in adult brain tissue by trauma may synergize with laminin ECM in NSPC niches to directly affect differentiation along the oligodendrocyte lineage^13^. Our data demonstrate the significant role mechanical forces such as stretch and ECM-integrin linkages play in dictating the lineage choice of NSPCs. This is the first reported case of a connection among active mechanical forces, specific ECM components, and oligodendrocyte differentiation.

A single active mechanical stretch stimulus was sufficient to induce a change in oligodendrocyte differentiation from NSPCs. Previous studies indicate that a single static stretch affects the function of multiple cell types, including MSCs, endothelial cells, and lung epithelial cells. Human MSCs exposed to static stretch exhibited increased proliferation as well as production and secretion of VEGF^11^. Rat coronary microvascular endothelial cells after static stretch increased proliferation, expression of VEGF receptor and vasculogenesis as demonstrated by formation of *in vitro* tubular structures^28^. Delivering a static stretch to human lung epithelial cells shifted their phenotype from alveolar type II to type I cells^29^. Taken together with our NSPC data, these studies show that a single static stretch activates signaling cascades affecting myriad cell types and functions.

NSPCs on laminin are able to sense an active stretch stimulus and translate the mechanical signal into a reduction in oligodendrocyte generation. ECM binding integrins are critical for mechanosensing in other systems and our data suggest the involvement of the laminin-binding α6 integrin in the stretch induced decrease in oligodendrocytes. Part of the mechanism by which static stretch affects NSPCs may involve a reduction in integrin clustering since the number of bound ligands per a given surface area is reduced by stretch and clustering is critical for integrin function^30^,^31^,^32^. Integrins are developmentally regulated in the oligodendrocyte lineage, and early precursors express laminin-binding α6β1 as well as the αV fibronectin-binding integrins αVβ1, αVβ3, and αVβ8^24^,^33^,^34^. While the cells in our study adhered well to fibronectin, this did not translate into an effect of stretch on oligodendrocyte differentiation (Fig. 4b). Much of the integrin mechanosensing literature has focused on fibronectin-binding integrins^35^, so little is known regarding the mechanical coupling of the laminin integrins to laminin. Thus, more studies will be necessary to test the role of the α6 laminin-binding integrin as a mechanosensor.

The specificity of NSPC stretch mechanosensing to laminin may be related to the *in vivo* function of these cells since *in vivo* cell niches are particularly rich in laminin ECM and laminin is associated with axons in the developing embryo^36^. For example, contact between developing axons and oligodendrocyte precursors and the associated ligation of precursor α6 integrins aids the further development of these cells into mature oligodendrocytes^36^. Thus, it is likely stem cell populations express mechanosensitive integrins that bind to the appropriate ECMs for that stem cell's niche in order to transduce *in vivo* mechanical signals and direct relevant physiological responses.

Since our experimental system enabled assessment of stretch and stiffness parameters, our data provide further evidence for substrate mechanics affecting NSPC fate although the stiffness range in our experiments is quite high compared to previous studies in the literature and was not meant to mimic a physiological range. We find increased differentiation of mNSPCs into oligodendrocytes and potentially neurons on membranes with stiffness ~1.6 MPa compared to membranes of stiffness ~10 kPa. In contrast, there was no significant effect on astrocyte generation in this stiffness range. Increasing substrate stiffness has been associated with decreased neuronal differentiation of adult rat hippocampal NSPCs, but within the stiffness range of 0.01–10 kPa^5^,^7^ which is well outside the range used in our model. Previous studies investigating NSPC differentiation into oligodendrocytes on substrates of varying stiffness have yielded conflicting results and used stiffness ranges outside those employed here. Oligodendrocyte differentiation from rodent NSPCs has been shown to increase with increasing substrate stiffness from 0.1–10 kPa, a range that does not reach the stiffness of the pre-stretched membrane in our studies^6^. However, a separate group reported decreases in oligodendrocyte differentiation from NSPCs in this same substrate stiffness range^7^. These discrepancies in differentiation pattern are likely due to varying substrate materials (polyacrylamide vs. methacrylamide chitosan) or cell source (hippocampus vs. forebrain SVZ NSPCs) in these studies. Additional studies with fewer variables will be needed to clarify the effects of stiffness on oligodendrocyte generation and the responses of NSPCs to a wide range of stiffnesses. Our data provide further evidence for mechanical factors affecting neural stem cell fate, showing substrates with stiffness in the ~1 MPa range can alter the differentiation of mNSPCs.

Our stiffness data were generated on pre-stretched membranes that were a control for the stiffness increase inherent in our stretched membranes. However, on pre-stretched membranes the laminin density may differ from that on unstretched membranes since cells are seeded on membranes after they are stretched and the laminin molecules coated on the membranes may be farther apart from each other after the application of the stretch. In order to control for this, we included the pre-stretched-then-coated condition, in which laminin is coated on the membrane after stretch to avoid perturbation by stretch. We assumed stretching the membrane prior to coating would not alter the binding affinity of laminin to the membrane surface. In support of this, we did not observe any difference in the total number of cells on the pre-stretched and pre-stretched-then-coated membranes, suggesting similar cell adhesion, proliferation, and survival on these two membranes, which would be consistent with comparable laminin coating. The fact that oligodendrocyte differentiation did not differ between cells on the pre-stretched and pre-stretched-then-coated membranes showed that the stiffness effects attributed to the pre-stretched membranes were not due to a reduction in laminin density.

Specific ECMs clearly impact the response of NSPCs to mechanical stresses. Thus, these warrant attention when designing biomaterial scaffolds for NSPC transplantation into the CNS. Scaffolds in the tissue will also transmit mechanical forces, which our data suggest will affect differentiation of the embedded cells. For this reason, materials for CNS applications should be designed with consideration of the mechanical environment that takes into account stiffness of the material, the mechanical stresses transplanted cells may encounter, and the ECM or integrin ligating component of the material.

Our findings identify the significant role active mechanical forces such as tensile strain play in NSPC lineage decisions and the involvement of ECMs in these choices. Knowledge of the impact of these forces on NSPC differentiation allows for better understanding of NSPC responses to physiological and pathological manipulations of CNS tissue.

## Methods

### J-Flex device to apply equibiaxial stretch

The J-Flex device utilizes rubber corks with circular Teflon disks adhered to their centers and applies a 10% static equibiaxial stretch to the membrane in the same manner as the Flexcell Tension System (Flexcell International Corporation) except instead of a negative pressure pulling down the membrane against the loading posts, the J-Flex device pushes the posts up against the membrane (Fig. 2a, b). The height of the Teflon disks regulates the percentage of applied strain to the membrane and it was determined that a disk height of 10.32 mm strains the membrane 10%. Measurements were made by applying markings to the membrane and quantitating their distance increase relative to one another using calipers once the device was in place. A 10% strain value was chosen as a relevant mechanical stimulus since this strain level has been applied to neural cells cultured on pliable membranes *in vitro* to model trauma and strains in this range were measured in models of surrogate brain material in human skulls subjected to rotational forces used to predict strain fields *in vivo*^37^,^38^,^39^.

### Cell culture and application of stretch

For the stretch experiments, NSPCs were isolated from the cerebral cortices of embryonic day 12.5 (E12.5) mice and passaged as non-adherent spheres for at least 1 passage before use. Cortical tissue from multiple embryos from each E12.5 litter was pooled, and subsequently cultured cells from an isolated litter was considered a biological repeat. Cells were seeded at 150,000 cells per membrane and 25,000 cells per 12 mm cover slip onto laminin (20 μg/mL) or fibronectin (10 μg/mL) coated Bioflex plates and glass cover slips as previously described^18^. Cells were left for 18 hours in proliferation conditions prior to differentiation for 3 days (neurons) or 7 days (astrocytes and oligodendrocytes). Proliferation media for E12.5 NSPCs is DMEM, 1 × B27, 1 × N2, 1 mM sodium pyruvate, 2 mM L-glutamine, 1 mM N-acetylcysteine, and growth factors 20 ng/mL EGF, 10 ng/mL FGF, and the cofactor 2 μg/mL heparin. Differentiation conditions utilize the same media without the inclusion of EGF, FGF, and heparin with an 100% differentiation media replacement done at 3 days. Adult rat hippocampal NSPCs were cultured as previously described^40^. NSPCs were cultured on unstretched, stretched, and pre-stretched membranes. Unstretched membranes contained cells that had no applied stretch stimulus, and stretched membranes had a 10% equibiaxial stretch applied 18 hours after cell seeding at the start of differentiation following the removal of growth factors or in mixed differentiation media (adult rat hippocampal NSPCs). The rate of equibiaxial strain application by the J-Flex device on the membranes in units of strain/sec was 20% (10% strain was reached in 0.5 ± 0.1 s). In the pre-stretched treatment, NSPCs were seeded onto membranes already experiencing a 10% equibiaxial stretch (applied after ECM coating), effectively eliminating any stretch stimulus to the cells. This group was included in order to distinguish substrate stiffness and stretch effects. In order to determine whether laminin on the pre-stretched membrane was altered by the stretch and thus contributing to the results observed on pre-stretched membranes, control membranes were stretched then coated with laminin to avoid perturbation of the laminin molecules (prestretched-then-coated treatment). In this condition, membranes were placed under stretch and then coated with laminin followed by cell seeding. The stiffness of the unstretched membrane is around 10 kPa while the stiffness of a membrane experiencing 10% equibiaxial stretch is close to 1.6 MPa as previously shown^41^. The elastic modulus of the membrane at 10% strain was measured using an Instron materials testing system at the University of California, Irvine and was found to be equal to 1.864 MPa. The slight discrepancy in stiffness between the Instron measurement and the value obtained in Ref. 41 can be attributed to the fact that the Instron system imposes a uniaxial strain as opposed to an equibiaxial strain. Cells were immunostained for the neuronal markers MAP2/DCX at 3 days differentiation and the astrocyte and oligodendrocyte markers GFAP and PDGFR-α/O4, respectively at 5–7 days differentiation since these time points provide quantifiable fields of cells for analysis as previously described^42^,^43^. PDGFR-α was used at 5 days on E12.5 cells since it is a marker for oligodendrocytes at an earlier stage in development. For adult rat hippocampal NSPCs, immunostaining for oligodendrocytes using O4 was done at 5 days since these cells displayed a higher propensity for oligodendrocyte differentiation than the E12.5 mNSPCs.

### Immunostaining and cell quantitation

Cells were fixed with 4% paraformaldehyde for 10 min as described previously^44^. Cells immunostained for cytoskeletal markers of neurons and astrocytes were treated with 0.3% Triton X-100 in PBS for 5 minutes prior to blocking for 1 hour in 5% bovine serum albumin (BSA) in phosphate saline buffer (PBS). Cells to be stained for surface markers of oligodendrocytes (O4 and PDGFR-α) were not treated with Triton X-100 prior to blocking. Prior to primary antibody staining, the silicone elastomer membranes from the Bioflex plates were cut out using a scalpel for easier manipulation. The region of the membrane used for analysis specifically excluded the edges where the teflon disk would create a bend in the membrane since this may represent a confounding topographical cue for the cells. Cells were then immunostained at 4°C overnight for neurons with mouse anti-MAP2 (Sigma M9942) and anti-DCX (Santa Cruz Biotechnology SC-8066 (c-18)) at 1:200, astrocytes with mouse anti-GFAP (Sigma G3893) at 1:200, and oligodendrocytes with mouse anti-O4 (R&D Systems MAB1326) at 1:100 and rabbit anti-PDGFR-α (Genetex GTX25460) at 1:200. Secondary antibodies (Alexa 488 and 555, Invitrogen A21206, A31570, A21432, A21426) were used at 1:200 at room temperature in the dark for 2 hours. Both primary and secondary antibodies were diluted in 1% BSA in PBS. Cell nuclei were counterstained for 1 minute with Hoechst 33342 at 1:500 in PBS. Coverslips and membranes with fixed cells were mounted with Vectashield (Vector Labs) and imaged using a Nikon Eclipse Ti microscope with a 10× or 20× objective and images were acquired using NIS element AR3.10 software.

The percentages of cells that had differentiated into neurons, astrocytes, and oligodendrocytes were calculated from 3–5 randomly selected fields per experiment. At least 3 independent experiments were performed with separate sets of cells (biological repeats), with more than 1500 cells quantitated and analyzed for each group. Total cell number was determined by counting all Hoechst-stained nuclei on membranes and glass. In order to assess generation of neurons, astrocytes, and oligodendrocytes from mNSPCs, strict criteria were applied. For neurons, cells expressing the neuronal markers MAP2 or DCX with neurites at least three times the length of the cell body were counted as neurons. For astrocytes, cells exhibiting a filamentous and cytoskeletal pattern of GFAP expression in the cytoplasm were counted as positive. In the case of E12.5 cortex derived mNSPCs, undifferentiated cells do not express GFAP, allowing for an accurate quantitation of astrocyte generation^44^. Oligodendrocytes were counted by clear expression of the extracellular markers O4 or PDGFR-α on cellular processes surrounding a cell nucleus. ImageJ was used to quantitate positively stained cells.

### Integrin blocking during migration

E12.5 mNSPCs spheres were allowed to adhere to coverslips that were pre-coated with laminin and pre-blocked with BSA (1% BSA in PBS) for 1 hour. Spheres were subsequently incubated with rat IgG2A isotype control antibody (clone 54447, 10 μg/mL; R&D Systems MAB006) or a function-blocking α6 integrin antibody (clone GoH3, 10 μg/mL; AbD Serotec MCA699EL) in proliferation medium. Spheres incubated in proliferation medium without antibody were also used as negative controls. The spheres were imaged at the time point of initial addition of integrin blocker or control and at 20, 90 minutes, 4, 16, and 24 hours. Radial migration of individual cells outward from the edge of the sphere was monitored and the distance traveled was measured at each time point.

### Integrin blocking during differentiation

E12.5 mNSPCs were plated onto laminin-coated unstretched or stretched membranes or glass coverslips that were pre-blocked with 1% BSA for 1 hour in order to reduce non-specific binding of antibody. Cells were seeded into the wells in proliferation media and allowed to adhere for 18 hours prior to switching to differentiation media. Adhered cells were incubated in α6 blocking antibody (clone GoH3, 10 μg/mL, AbD Serotec MCA699EL), IgG2A isotype control antibody (clone 54447, 10 μg/mL, R&D Systems MAB006), or no antibody for 2 hours in differentiation media to allow for antibody infiltration onto the ventral surface of the cells, followed by the application of 10% equibiaxial stretch in the respective stretch groups. Membranes were fixed and immunostained for the oligodendrocyte marker O4 after 7 days of differentiation, with a 100% differentiation media replacement done at 3 days.

### Statistical Analysis

All statistical analyses utilized a one-way single factor ANOVA to compare two samples and utilized data from three or more independent biological repeats.

## Author Contributions

J.A., M.M.P., J.L.N., F.T. and L.A.F. designed experiments; J.A. performed experiments and assays; L.P.M. performed dissections to obtain cells used in experiments; J.A. and J.C.E. designed and built the J-Flex device; J.A. analyzed data; J.A. and L.A.F. wrote the manuscript; L.A.F. supervised the project; All authors edited and approved the final manuscript.

## Supplementary Material

Supplementary Information Supplementary Information

## Figures and Tables

**Figure 1:**
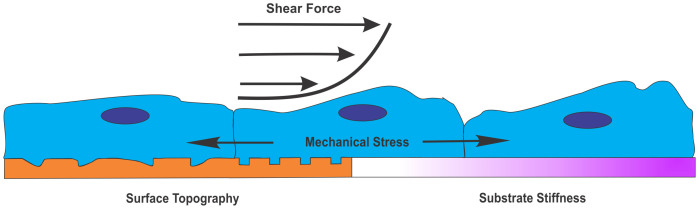
Physical regulators of stem cell behavior. Stem cells experience a variety of physical cues within the natural microenvironment that can have significant effects on survival, proliferation, differentiation, and gene expression^2^. Examples of these mechanical cues include shear force from fluid, mechanical stress from cell-cell interactions and cell movements, and surface topology and substrate stiffness through components of the ECM and surrounding cells.
