# Supplementary material for: Static stretch affects neural stem cell differentiation in an extracellular matrix-dependent manner
Source: Sci Rep. 2015 Feb 17;5:8499. doi: 10.1038/srep08499 (PMC4330529; doi:10.1038/srep08499)
Supplement: Supplementary Information [file srep08499-s1.pdf]

Supplementary Information

Static stretch affects neural stem cell differentiation in an extracellular matrix-dependent manner

Janahan Arulmoli[1,2], Medha M. Pathak[3], Lisa P. McDonnell[2,4], Jamison L. Nourse[2,4], Francesco Tombola[3], James C. Earthman[1,5], Lisa A. Flanagan[1,2,4*]

[1]Department of Biomedical Engineering, University of California, Irvine, 3210 Natural Sciences II, Irvine, CA 92697-2715, USA

[2]Sue & Bill Gross Stem Cell Research Center, University of California, Irvine, 845 Health Sciences Road 3200 Gross Hall, Irvine, CA 92697-1705, USA

[3]Department of Physiology & Biophysics, University of California, Irvine, D340 Medical Sciences I, Irvine, CA 92697-4560, USA

[4]Department of Neurology, University of California, Irvine, 200 South Manchester Avenue Suite 206, Orange, CA 92868-4280, USA

[5]Department of Chemical Engineering and Materials Science, University of California, Irvine, 916 Engineering Tower, Irvine, CA 92697-2575, USA

*Correspondence should be addressed to L.A.F. (lisa.flanagan@uci.edu)

Supplemental Figures:

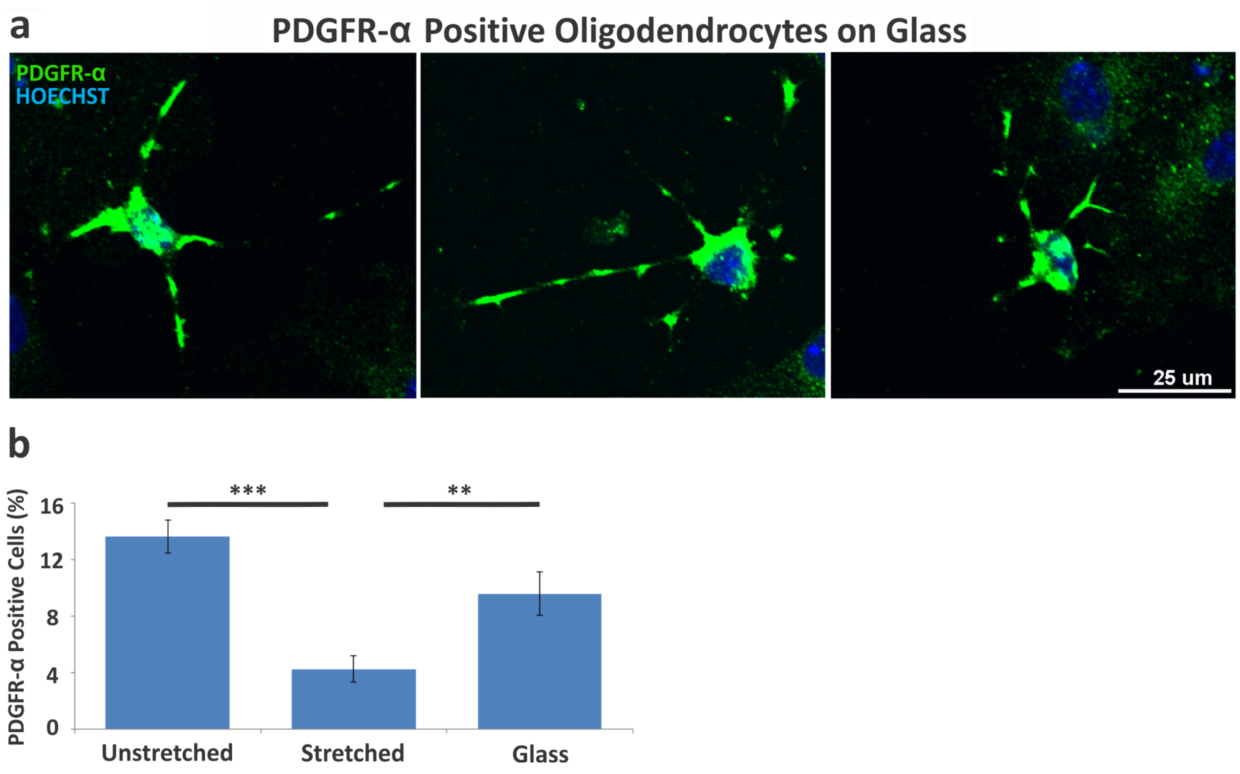

Supplemental Figure S1 | Stretch inhibits mNSPC differentiation into PDGFR-α-positive oligodendrocytes. a, Images of E12 mNSPCs differentiated on glass showing cell surface oligodendrocyte marker PDGFR-α co-stained with nuclear DNA marker Hoechst. **b,** Static stretch reduces oligodendrocyte differentiation (cells on stretched compared to unstretched membranes). PDGFR-α recognizes oligodendrocytes at an earlier stage of differentiation than O4, so the percentage of cells identified with this marker is higher than that recognized by the more mature marker O4 as shown in Figure 3a. P=1.15E-06 (unstretched vs. stretched). P=0.005 (glass vs. stretched). **P<0.01, ***P<0.001. Error bars represent SEM. N=3 independent biological repeats.

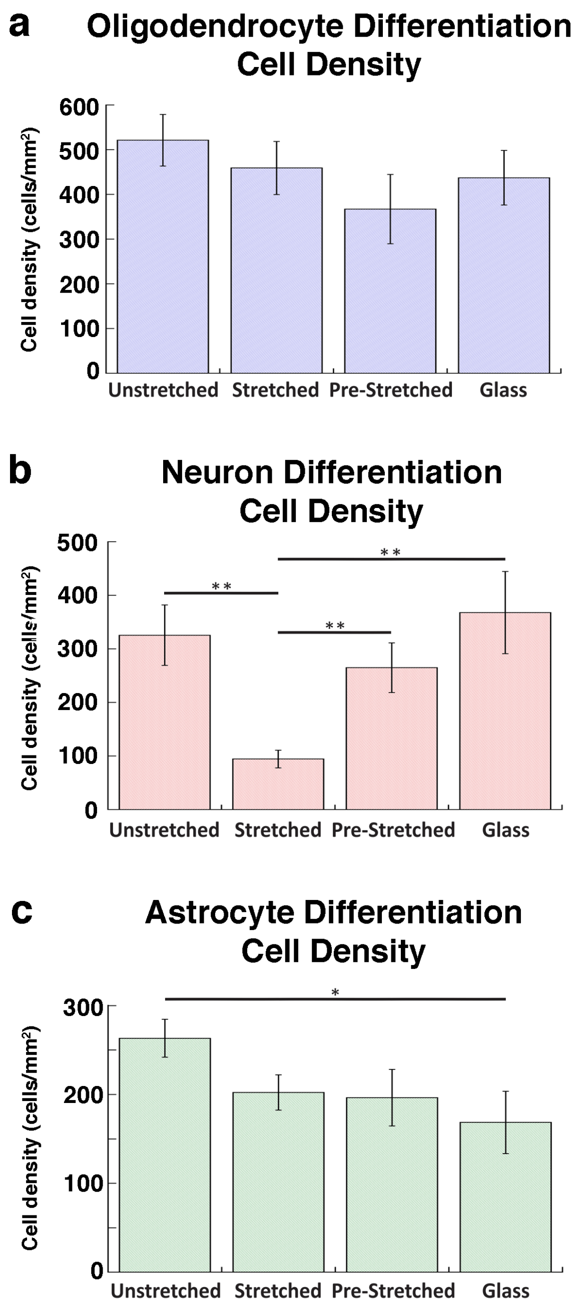

Supplemental Figure S2 | Cell density quantitation of differentiated mNSPCs. The average cell density in cells/mm^2 is shown for cells on unstretched, stretched, and pre-stretched membranes and glass for **a,** oligodendrocyte **b,** neuron, and **c,** astrocyte cell differentiation experiments matching those shown in Figure 5. *$P<0.05$, **$P<0.01$. Error bars represent SEM. N=3 independent biological repeats.

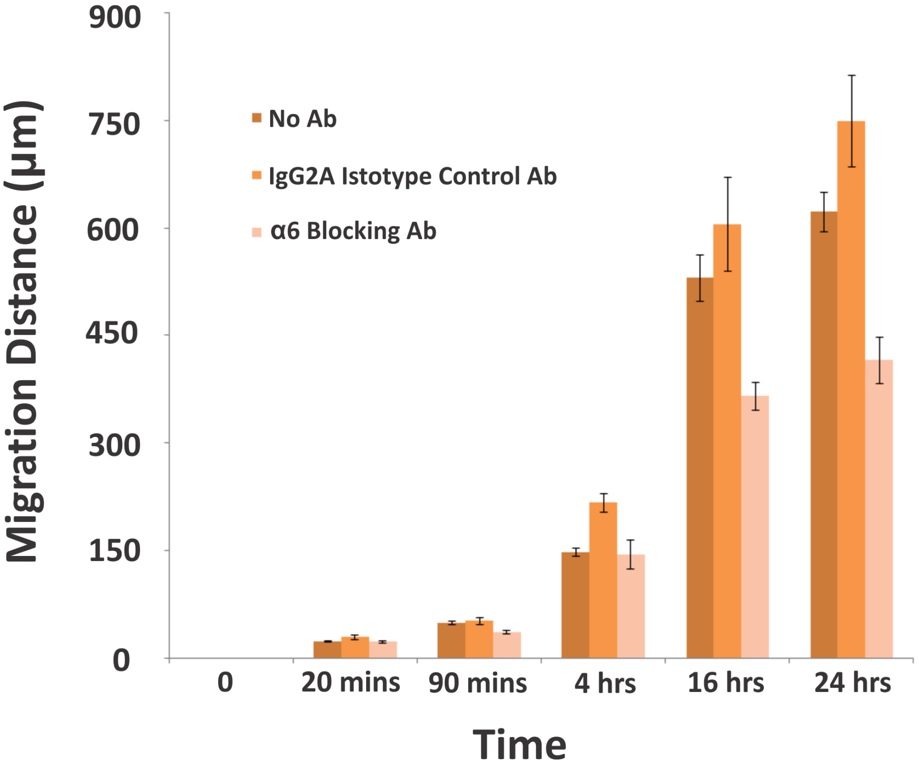

Supplemental Figure S3 | E12 mNSPCs express functional α6 integrin. mNSPC migration out of spheres over 24 hours during incubation with no antibody (No Ab), IgG2A isotype control antibody, or α6 integrin function-blocking antibody. Perturbing α6 integrin using a function-blocking antibody decreases migration of mNSPCs on laminin substrates, indicating these cells express functional cell surface α6 integrin. Error bars represent SEM.

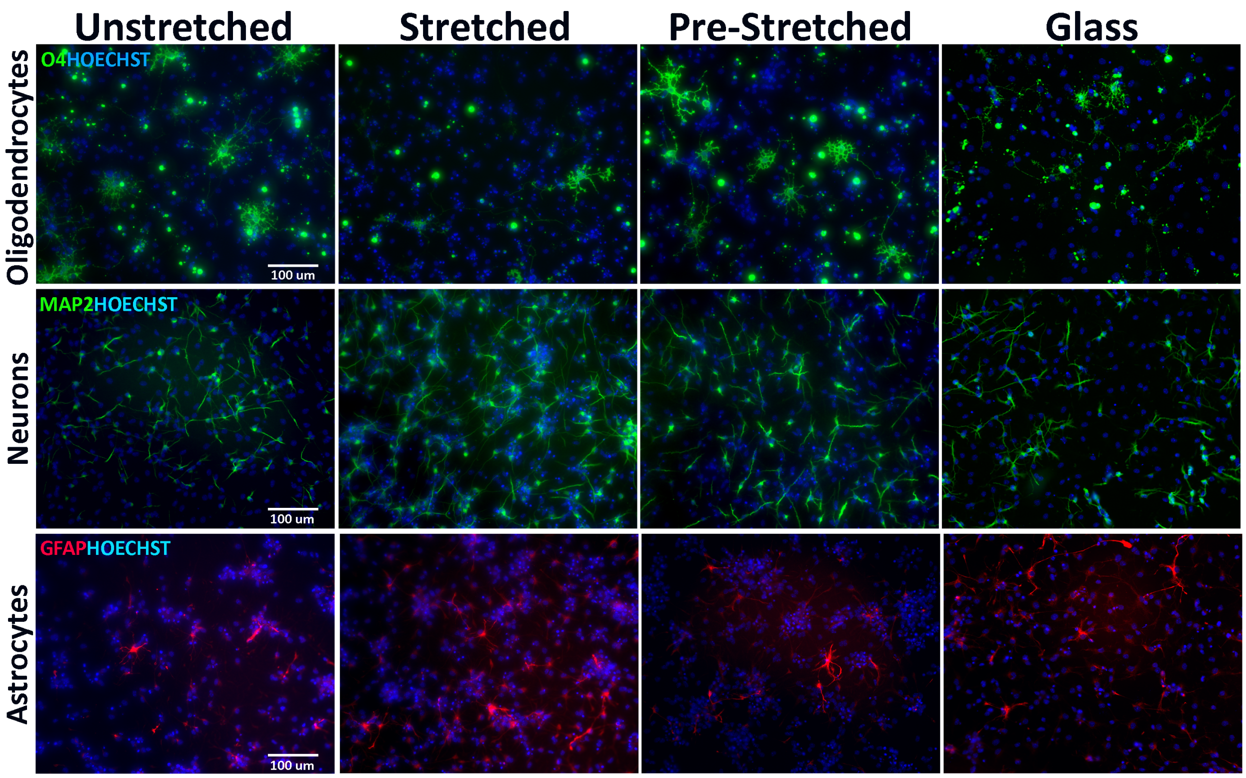

Supplemental Figure S4 | Immunocytochemistry of mNSPC differentiation. Images of E12 mNSPCs differentiated on unstretched, stretched, and pre-stretched membranes and glass showing nuclear DNA marker Hoechst co-stained with cell surface oligodendroglial marker O4 (top), cytoskeletal neuronal marker MAP2 (middle), and cytoskeletal astrocytic marker GFAP (bottom).

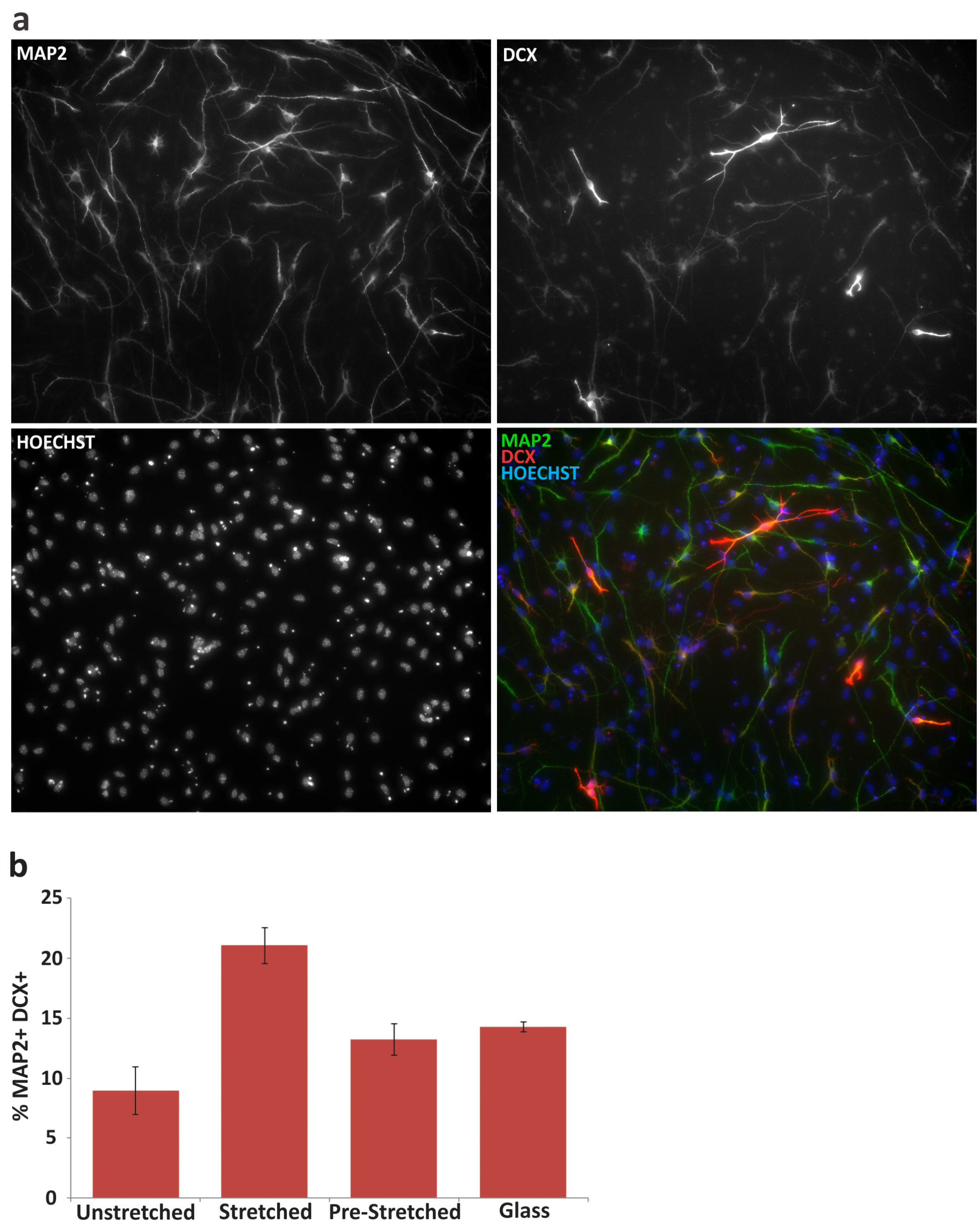

Supplemental Figure S5 | Immunocytochemistry of neurons differentiated from mNSPCs using multiple markers. a, Images of E12 mNSPCs differentiated on an unstretched membrane showing neuronal markers MAP2 (top left) and DCX (top right) with nuclear DNA marker Hoechst (bottom left) and the co-stain (bottom right). **b,** Quantitation of neurons using two markers (MAP2 and DCX) shows a pattern similar to that presented in Figure 5 with MAP2

alone; more neurons are generated from mNSPCs on stretched or pre-stretched membranes and glass than those on unstretched membranes. There was no statistical analyses performed since N=1 independent biological repeat for these data. Error bars represent SEM of multiple quantified fields.
